# Structure-Function Relationship of a Plant NCS1 Member – Homology Modeling and Mutagenesis Identified Residues Critical for Substrate Specificity of PLUTO, a Nucleobase Transporter from Arabidopsis

**DOI:** 10.1371/journal.pone.0091343

**Published:** 2014-03-12

**Authors:** Sandra Witz, Pankaj Panwar, Markus Schober, Johannes Deppe, Farhan Ahmad Pasha, M. Joanne Lemieux, Torsten Möhlmann

**Affiliations:** 1 Department of Plant Physiology, University of Kaiserslautern, Kaiserslautern, Germany; 2 Membrane Protein Disease Research Group, Department of Biochemistry, University of Alberta, Edmonton, Alberta, Canada; 3 Catalysis Research Center, King Abdullah University of Science and Technology (KAUST), Thuwal, Kingdom of Saudi Arabia; Leibniz-Institute for Vegetable and Ornamental Plants, Germany

## Abstract

Plastidic uracil salvage is essential for plant growth and development. So far, PLUTO, the plastidic nucleobase transporter from *Arabidopsis thaliana* is the only known uracil importer at the inner plastidic membrane which represents the permeability barrier of this organelle. We present the first homology model of PLUTO, the sole plant NCS1 member from Arabidopsis based on the crystal structure of the benzyl hydantoin transporter MHP1 from *Microbacterium liquefaciens* and validated by molecular dynamics simulations. Polar side chains of residues Glu-227 and backbones of Val-145, Gly-147 and Thr-425 are proposed to form the binding site for the three PLUTO substrates uracil, adenine and guanine. Mutational analysis and competition studies identified Glu-227 as an important residue for uracil and to a lesser extent for guanine transport. A differential response in substrate transport was apparent with PLUTO double mutants E227Q G147Q and E227Q T425A, both of which most strongly affected adenine transport, and in V145A G147Q, which markedly affected guanine transport. These differences could be explained by docking studies, showing that uracil and guanine exhibit a similar binding mode whereas adenine binds deep into the catalytic pocket of PLUTO. Furthermore, competition studies confirmed these results. The present study defines the molecular determinants for PLUTO substrate binding and demonstrates key differences in structure-function relations between PLUTO and other NCS1 family members.

## Introduction

Salvage of nucleosides and nucleobases represents an alternative to the *de novo* synthesis of nucleotides. Hereby, energy and also high valuable constituents of purine and pyrimidines for plant development namely phosphate and nitrogen are preserved. It was shown that salvage of uracil and of uridine in plastids is essential for growth and development of Arabidopsis seedlings [1], [2]. Furthermore, stimulation of the pyrimidine salvage pathway in potato leads to an increase in biosynthetic performance and starch contents in tubers [3].

Our recent results indicate that pyrimidine *de novo* synthesis is finalized in the cytosol and consequently pyrimidines must be initially imported into plastids to serve for the synthesis of nucleic acids in this compartment by salvage reactions [4]. Whereas no nucleoside transport system has been described for plastids so far, a corresponding nucleobase transporter was identified. PLUTO, the plastidic nucleobase transporter is the sole member of the NCS1 (Nucleobase:Cation Symporter1) protein family in Arabidopsis [4], [5]. Other plant species like rice (*Oryza sativa*), maize (*Zea mays*), wine (*Vitis vinifera*) or poplar (*Populus trichocarpa*) similarly only harbor one *PLUTO* homolog. However, in the grass species *Brachypodium distachyon* two *PLUTO* homologs are present [6]. Recently, a PLUTO homolog from the single cell algae *Chlamydomonas reinhardtii* was identified, mediating transport of adenine, guanine, uracil and allantoin. This shows that solute specificity of plant NCS1 members arose early in plant evolution [7].

PLUTO plant homologs show an N-terminal extension counted from the beginning of the first transmembrane span of about 80 amino acids. From the species mentioned above, only *P. trichocarpa* exhibits a shorter N-terminus (38 amino acids), whereas Arabidopsis PLUTO exhibits a much longer N-terminal extension of 134 amino acids [6]. Such sequence extensions are typical for eukaryotic transport proteins [8] and in fact prokaryotic NCS1 family members like MHP1, HYUP and PUCI do not show such extensions [4]. Heterologous expression of the full length *PLUTO* Arabidopsis gene in *Escherichia coli uraA* insertion mutants demonstrated PLUTO facilitates high affinity nucleobase transport. The apparent affinities of uracil, adenine and guanine were determined with 16.4, 0.4, and 6.3 μM, respectively [4]. While full length PLUTO can transport nucleobases, protein synthesized from a second putative start codon at position +97, almost lacking the complete N-terminal extension was not functional in the *E. coli* system and showed no targeting to chloroplasts in transient expression studies [4]. However, in the EMBL database PLUTO protein is annotated as N-terminal truncated ORF (Q93726) and was shown to function better in a yeast expression system, compared to the longer ORF [5]. At present it is unknown whether both protein variants exist in Arabidopsis.

Plastids are plant specific organelles fulfilling a multitude of biochemical reactions such as photosynthesis, nitrogen and sulfur assimilation, starch, fatty acid and amino acid synthesis. For the necessary exchange of pathway intermediates transport proteins are located in the inner envelope membrane of plastids, which serves as a permeability barrier in these organelles. Amongst these, transporters for triose-phosphates, sugars and phosphate have been characterized (for review see [9]). In addition to PLUTO further transporters catalyzing the exchange of intermediates of nucleotide metabolism exist in plastids. These facilitate the exchange of ATP against ADP (NTT, [10], [11]) and the net exchange of ATP, ADP and AMP (BT1, [12]).

NCS1 proteins like PLUTO are predicted to contain 12 transmembrane spans and are also known as purine-related transporters. Being symporters they generally transport purines using a co-transport with protons. Apart from plants they can be found in archaea, bacteria, yeast and fungi [13] (www.tcdb.org). Well-characterized NCS1 proteins with marked homology to PLUTO are the uracil transporter FUR4 and the uridine transporter FUI1 from *Saccharomyces cerevisiae* [14], [15], the allantoin transporter DAL4 from *Saccharomyces cerevisiae* [16], [17] and FCYB from *Aspergillus nidulans*. The latter was characterized as purine-cytosine transporter and analyzed with respect to structure–function relationships recently [8].

The first structural information about the NCS1 transporters came through MHP1, the benzyl-hydantoin transporter from *Microbacterium liquefaciens.* The MHP1 crystal structure revealed a monomer organized into an alpha-helix bundle with 12 trans-membrane (TM) helices that form a deep internal cavity open on the periplasmic side with the substrate binding site located at the center of the protein cavity. The 12 TMs are arranged in two repeating halves (TMs 1 to 5 and TMs 6 to 10), connected by an 18-residue loop and followed by two additional TMs. Quite remarkably, MHP1 structure has been solved at different stages along the transport cycle, inward facing, occluded and outward facing states, which provides the structural basis for substrate transport [18]. By the analysis of these three MHP1 conformations coupled with molecular dynamics simulations a transport mechanism for MHP1 has been proposed [19]. The conformational transition from the outward state to the inward state results from rigid body motion of the TM helices 3, 4, 8 and 9 relative to the rest of the protein. This provides the molecular basis for the alternative access mechanism of substrate transport, which could be possibly applicable to all the members of NCS1 family. This “rocker switch” mechanism ensures “uphill” transport of substrate against its concentration gradient when it is coupled to energetically favorable “downhill” transport of the driving ion Na^+^.

The MHP1 complex with benzyl hydantoin along with other conformations provides insight into the substrate transport mechanism. However, details about the substrate binding sites of NCS1 family members for other known substrates including purines, cytosine and uracil are lacking. Despite a common predicted structural fold with the NCS1 family, these transporters share divergent amino acid sequences which may lead to variations within the substrate binding sites and transport of a wide variety of substrates. In this study, we aim to address this issue by identifying critical residues involved in the binding of the physiological substrates namely adenine, guanine and uracil within the sole plant NCS1 family member PLUTO. We provide an experimentally validated three-dimensional model of the binding site in PLUTO using mutational analysis of PLUTO paired with structural-activity data for the substrates. Validation of binding models was performed based on the mutational dataset of PLUTO point mutants describing the contribution of residues in the putative substrate binding pocket. Together with results from substrate competition studies mechanistic insights into PLUTO transport function were obtained.

## Materials and Methods

### Heterologous expression of PLUTO and site directed mutants in *E. coli* JD23420

For the uptake experiments, PLUTO was heterologously expressed in an *Escherichia coli* transposon insertion strain lacking the endogenous uracil permease uraA (JD23420), obtained from the National Institute of Genetics (Shizuoka, Japan; http://www.shigen.nig.ac.jp/ecoli/strain/top/top.jsp). PLUTO and site directed mutants were cloned in the bacterial expression vector pTAC-MAT-Tag-2 (Sigma Aldrich, Deisenhofen, Germany) as previously described [4]. After the transformation of the cells with the *PLUTO* expression plasmids, the cells were grown at 37°C and 170 rpm in YT medium containing ampicillin and kanamycin. The expression of *PLUTO* and site directed mutants was initiated by the addition of isopropyl β-D-thiogalactopyranosid (IPTG) with a final concentration of 0.02% at an OD_600_ of 0.5. The cells were grown for 2 h after induction, collected by centrifugation for 5 min at 4500 *g* and 4°C and resuspended with potassium phosphate buffer (50 mM, pH 7.0) to an OD_600_ of 5. The cells were stored on ice until use.

### Site directed mutagenesis

Site directed mutagenesis was achieved by using the QuickChange II-E Site directed mutagenesis kit (Stratagene) according to the manufacturer's instructions. Primers used are listed in Table S1.

### Nucleobase uptake experiments with PLUTO and site directed mutants

For the uptake experiments with radiolabelled nucleobases, 100 μl IPTG-induced cells and uninduced cells used as negative controls were added to the same volume of preheated potassium phosphate buffer (50 mM, pH 7.0, 37°C) containing radiolabelled nucleobases ([^14^C]-uracil, -guanine and –adenine, 1012 Bq mol^−1^). The uptake of nucleobases with concentrations of 20 μM was performed at 37°C and permanent shaking at 200 rpm. For the analysis of PLUTO mutants the cells were incubated for 5 min (2 min for competition studies) and the uptake was terminated by vacuum filtration through a membrane filter (0.45 μm pore size; Whatman). The cells were washed three times with 1 ml ice cold potassium phosphate buffer (50 mM, pH 7.0) to remove unimported nucleobases and the radioactivity was quantified in a Packard Tricarb 2500 scintillation counter after the addition of 4 mL scintillation cocktail (Roth). For the competition studies, all competitors were added with ten-fold excess (200 μM) to the uptake medium containing 20 μM radiolabelled nucleobases. To show that PLUTO and PLUTO mutants have no marked differences in protein amount, membranes from sonified *E. coli* cells were collected by sequential centrifugation, spotted on hybridization membranes and probed with a MAT-Tag specific antibody (Figure S1).

### Construction of the Sequence Alignment

Multiple alignments of protein sequences were performed using ClustalW [20] and visualized with Genedoc. Sequences used are as follows: *Arabidopsis thaliana* PLUTO (At5g03555, AED90625), *Microbacterium liquefaciens* MHP1 (2JLN_A) *Aspergillus nidulans* FCYB (GI: 169798762), PLUTO homologs from *Oryza sativa* (Os02g44680), Zea mays (Zm362848), *Vitis vinifera* (GSVIVT01033705001), *Populus trichocarpa* (Pt0006S12110), and *Brachypodium distachyon* (Bradi3g51350).

### Homology modeling

The three-dimensional model of the PLUTO full length sequence was built using crystal structures of MHP1 in the outward-facing open conformation (PDB ID 2JLN) as a template. Structural modeling was performed using the I-TASSER server based on *ab initio*/threading method [21]. The server predicted 5 models of PLUTO and the best model was selected based on C-score (0.59). C-score is a confidence score for estimating the quality of predicted models by I-TASSER. It is calculated based on the significance of threading template alignments and the convergence parameters of the structure assembly simulations. Quality control analysis by the PROCHECK program identified 90% residues of the model in the most favorable regions of the Ramachandran plot [22]. Only five glycine and nine non-glycine, non-proline residues (1% and 1.8% of the primary structure) were in the forbidden region of the Ramachandran plot. For the evolutionary conservation profile of the PLUTO core protein (TM1-TM10) the conservation score was calculated based on ClustalW matrix by Conserv Server [23].

### Molecular dynamics simulations

The initial homology model was subjected to protein preparation tool implemented in Schrodinger suite 2012. The N-terminal part of PLUTO which does not align to MHP1 was removed. In summary the hydrogen atoms were added and the protonation states of histidine residues were optimized based on pKa calculation. A restrained minimization was performed using Impact MM engine implemented in Schrodinger suite 2012. The molecular dynamics (MD) simulation was performed using Desmond v.3 software. The protein structure was embedded into a 1-palmitoyl-2-oleylphosphatidylcholine (POPC) lipid bilayer. The length and width of lipid box was 105 Å×117 Å. The system was solvated using TIP4P explicit water and neutralized by adding 150 mM sodium ions. After membrane insertion and solvation of the system, the lipids were re-equilibrated for 10 ns. The MHP1 template available in OPM database was used to set orientation of protein in the membrane [24]. At last, a total of 20 ns MD simulations were performed on the system under a constant temperature of 300 K and a constant pressure of 1 bar.

### Molecular Docking

The protein structure obtained after homology modeling and MD simulations were employed in docking calculation performed with Glide 5.0 (Schrodinger, LLC). All three substrates were minimized using the OPLS-2005 force field by Macro Model software [25]. Details of the algorithm are available in the GLIDE documentation. Briefly, GLIDE proprietary conformational expansion and exhaustive search of the binding site produce a multitude of ligand poses, which undergo an initial refinement, energy minimization on a precomputed grid, and a final scoring and ranking. GLIDE uses proprietary scoring functions that are variations of the Chem Score 43 empirical scoring function and the OPLS-2005 force field to compute van der Waals and electrostatic grids for the receptor. The final ligand binding poses were ranked according to a computed Emodel score that encompasses the grid score, the proprietary Glide score, and the internal energy strain. The substrates were docked into the receptor site using GLIDE SP (standard precision) mode. A total of 50 docked conformers for each substrate were obtained. The best pose for each ligand was selected using total energy as a primary criterion and the experimental knowledge as a secondary criterion.

## Results

### Identification of critical residues for substrate binding through sequence homology

**Figure 1:**
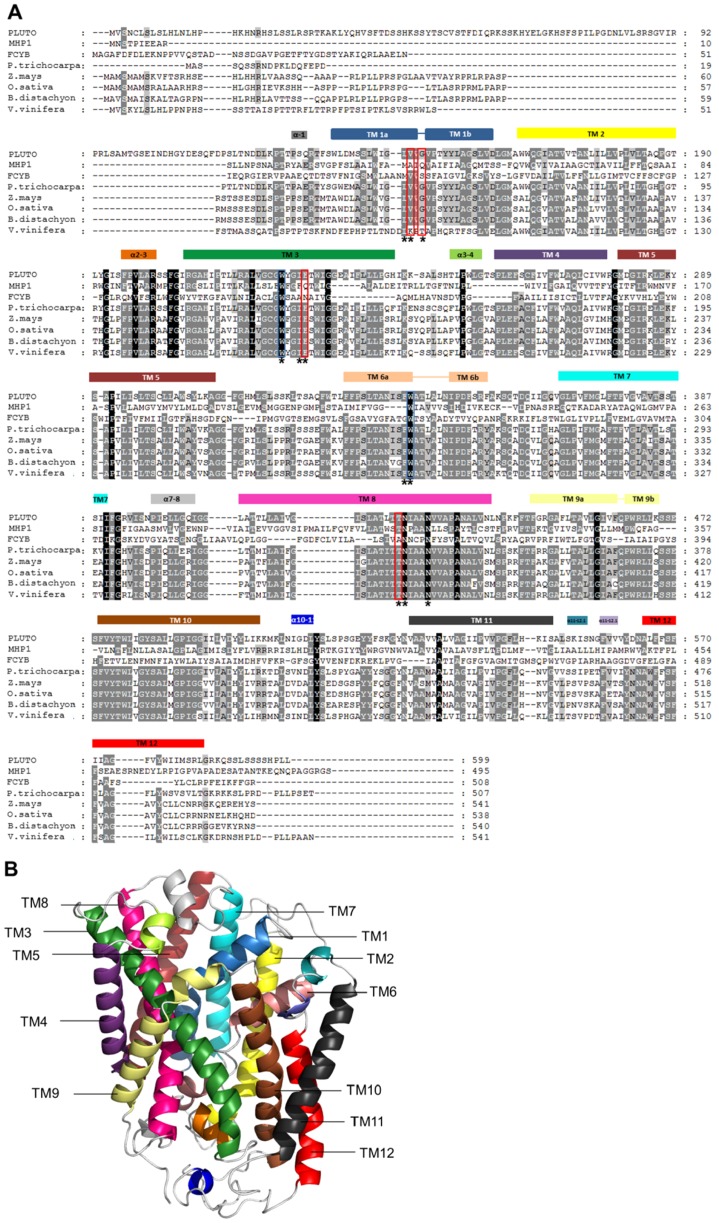
Alignment of PLUTO with other NCS1-type protein sequences from different organisms and PLUTO homology model. (A) NCS1 proteins from *Arabidopsis thaliana* PLUTO (AED90625), *Microbacterium liquefaciens* MHP1 (2JLN_A) *Aspergillus nidulans* FCYB (GI: 169798762), and PLUTO homologs from *Oryza sativa* (Os02g44680), *Zea mays* (Zm362848), *Vitis vinifera* (GSVIVT01033705001), *Populus trichocarpa* (Pt0006S12110), *Brachypodium distachyon* (Bradi3g51350) were aligned with ClustalW (www.ebi.ac.uk). The residues are shown in white, gray or black color according to their conservation mode (black for highly conserved residues). Residues marked with asterisks were mutated in the course of this work. Residues marked with red boxes are directly involved in PLUTO substrate binding. Blue boxes indicate two Trp residues which are highly conserved among NCS1 proteins and probably exhibit a function in stabilizing the protein-substrate complex with weak pi-stacking interactions. (B) PLUTO homology model with marked TMs. A three-dimensional model of PLUTO was built using I-TASSER server based on structural information of MHP1. The TMs are marked in colors according to the alignment in Figure 1A. The N-terminus of PLUTO was removed for better visualization.

PLUTO (At5g03555), identified as plastidic nucleobase transporter in Arabidopsis [4], belongs to the Nucleobase:Cation Symporter1 (NCS1) family of transport proteins. Further NCS1 members are FUR4, FUI1, and DAL4 from *Saccharomyces cerevisiae*, HYUP from *Arthrobacter aurescens*, PUCI from *Bacillus subtilis*, and MHP1 from *Microbacterium liquefaciens*. Among these sequences 34 amino acid residues are highly conserved including six residues known to be involved in substrate binding based on the available crystal structure of MHP1 [26]. From sequence alignment with MHP1, FCYB and six further sequences from plants (Figure 1A), we observe that four of these residues, Trp-223, Trp-342, Asn-426 and Asn-430, relative to PLUTO are conserved in all sequences, whereas two, Gly-147 and Glu-227 differ. Gly-147 of PLUTO is equivalent to Gln-42 in MHP1 and Glu-227 correspondingly aligns to Gln-121 [4]. All six named positions are conserved in all plant species shown in the alignment except for *Vitis vinifera* possessing a G147T exchange (Figure 1A). Based on the information from the alignments we aimed to gain insight into the structure function relationship of nucleobase binding and transport mediated by PLUTO. To do so, both non conserved amino acid residues in PLUTO, Gly-147 and Glu-227 which are involved in substrate binding in MHP1 were mutated and the protein was subsequently tested for transport activity after expression in *E. coli* cells as previously described [4]. Heterologously expressed PLUTO carries a C-terminal MAT-Tag (Sigma Aldrich) used to identify the protein in western blots (Figure S1). The tag does not negatively influence transport activity of PLUTO. In contrast, the background of uninduced controls was lower when PLUTO was synthesized with tag (Figure S2).

### Functional studies with PLUTO mutants

**Figure 2:**
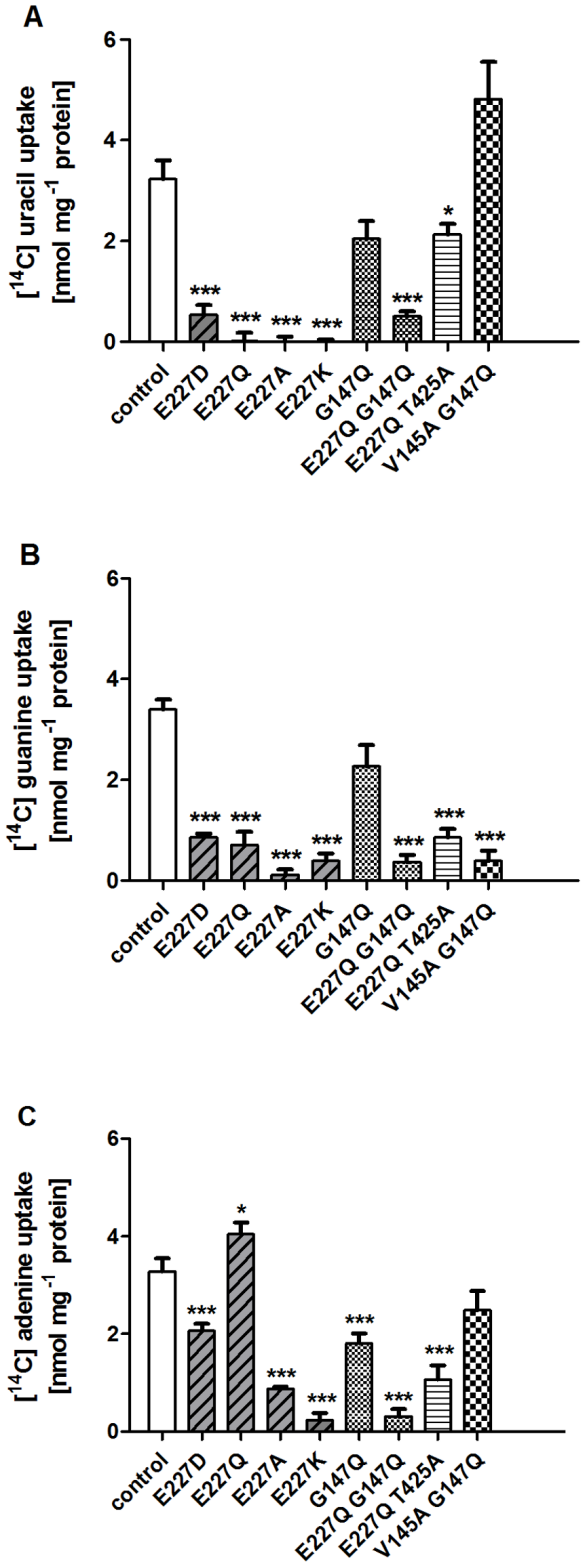
Functional analysis of PLUTO mutants. Radiolabeled nucleobase uptake studies with all substrates of PLUTO, namely uracil, guanine and adenine were performed after heterologous PLUTO expression in *E. coli* cells lacking the endogenous uracil transporter uraA. Uptake was measured for 5 min as previously described in [3] with nucleobase concentrations of 20 μM. The data represent the mean of net uptake rates of at least three independent experiments ± SE. The asterisks indicate significant differences between PLUTO mutants and the control based on Student's t-test (*  =  p<0,05; ***  = p<0,005).

In the mutagenesis approach, first, Glu-227 was exchanged to an aspartate residue also carrying a negative charge. E227D showed markedly reduced transport of uracil and guanine (Table 1, Figure 2). Whereas wild type PLUTO protein catalyzed import of 3.7±0.28 nmol [^14^C]-uracil mg^−1^ protein (20 μM) into induced *E. coli* cells, uninduced cells, used as negative controls, only imported 0.5±0.11 nmol [^14^C]-uracil mg^−1^ protein within 5 min incubation leading to a net import of 3.2 nmol [^14^C]-uracil mg^−1^ protein (Figure 2A). In E227D expressing cells uracil transport was reduced to 0.53 nmol mg^−1^ protein. Guanine transport (20 μM) was reduced to 0.86 nmol mg^−1^ protein relative to controls which accounted for 3.4 nmol mg^−1^ protein (Figure 2B). In contrast, adenine transport (20 μM) was only slightly reduced to 2.1 nmol mg^−1^ protein representing 64% of the transport rates of controls (Figure 2C). The calculated K_M_ values in this mutant were 5.18, 3.70 and 0.97 for uracil, guanine and adenine, respectively (Table 2). In the next step negatively charged glutamate at position 227 was mutated to an uncharged glutamine (E227Q), a smaller and uncharged alanine (E227A), or to a positively charged lysine, E227K. In any of these cases uracil transport activity was completely extinguished (Figure 2A) and guanine transport was reduced to 21%, 4% and 11%, respectively (Figure 2B). However, adenine transport even increased in E227Q by 21% (Figure 2C) whereas the affinity remained nearly unchanged (Table 2). E227A and E227K mutations led to residual adenine transport of 26% and 7% compared to controls, respectively (Figure 2C). Wild type and mutated PLUTO expressing cells showed no marked differences in protein amount. For this, membranes from sonified *E. coli* cells were collected by sequential centrifugation, spotted on membranes and probed with a MAT-Tag specific antibody (Figure S1). No marked differences in protein amount were observed.

**Table 1:** Nucleobase transport activity of different PLUTO mutants.

Mutant	Uracil transport	Guanine transport	Adenine transport
E227Q	**-**	**+**	**+++**
E227A	**-**	**-**	**+**
E227K	**-**	**-**	**-**
E227D	**-**	**+**	**++**
G147Q	**++**	**++**	**++**
G147K	**+++**	**+++**	**+++**
E227Q G147Q	**-**	**-**	**-**
E227Q T425A	**++**	**+**	**+**
E227Q V145A	**+++**	**+++**	**++**
V145A	**+++**	**++**	**++**
V145A G147Q	**+++**	**-**	**++**
T425A	**+++**	**+++**	**+++**
W342A	**+++**	**+++**	**+**
W223A	**++**	**++**	**++**
L144A	**+++**	**+++**	**+++**
F341A	**+++**	**+++**	**++**
N426A	**++**	**++**	**++**
N430A	**++**	**++**	**++**
I226A	**++**	**++**	**+++**

The transport of uracil, guanine and adenine, 20 μM each, was measured after heterologous *PLUTO* expression in *E. coli* cells lacking the endogenous uracil importer uraA. Transport was corrected for uptake into uninduced cells. Transport function above 80% of control is indicated with “**+++** ”, between 80% and 50% with “**++**”, between 50%and 20% with “**+**” and below 20% with “**-**”.

**Table 2:** Substrate affinity of PLUTO and PLUTO mutants.

Mutant	Uracil	Guanine	Adenine
	K_M_ ± SE [μM]	K_M_ ± SE [μM]	K_M_ ± SE [μM]
Control	16.43 (±5.10)	6.29 (±2.52)	0.38 (±0.16)
E227Q	-	-	0.22 (±0.05)
E227A	-	-	0.21 (±0.05)
E227D	5.18 (±1.21)	3.70 (±0.72)	0.97 (±0.21)
G147Q	36.07 (±2,32)	4.55 (±1,00)	0.68 (±0.20)
V145A G147Q	20.15 (±3.73)	-	0.81 (±0.17)

K_M_- values are provided for uracil, guanine and adenine of different PLUTO mutants ± standard error (SE) and were determined after heterologous *PLUTO* expression in *E. coli* cells lacking the endogenous uracil importer uraA. Transport was corrected for uptake into uninduced cells.

Mutation of the second site of interest based on the sequence comparison between PLUTO and MHP1, Gly-147 was also carried out. Adenine transport was impaired by 45% when Gly-147 was mutated to glutamine G147Q, whereas uracil and guanine transport were not significantly altered (Figure 2 A-C). The affinity towards uracil in this mutant slightly decreased whereas affinities for adenine and guanine remained nearly unchanged (Table 2). However, in the double mutant E227Q G147Q, which resembles the MHP1 structure with respect to the identified substrate binding site residues, transport of all the identified substrates uracil, guanine and adenine was reduced to 16% or below (Figure 2 A-C, Table 1). In contrast to the single mutation E227Q also adenine was no longer transported although the protein amount in *E. coli* membranes was not changed compared to controls and this holds true for all other mutants described in this paragraph (Figure S1).

### PLUTO structural model

To obtain further insight into the PLUTO structure-function relationship, we took advantage of the close sequence homology between PLUTO and MHP1 and developed a homology model based on the outward facing conformation of MHP1 [18] (Fig 1B, 3A). The overall protein sequence homology between MHP1 and PLUTO (26% sequence identity and 48% sequence similarity) is adequate for sustaining a theoretical model [21]. The model building was performed using I-TASSER and 5 top-scoring homology models of PLUTO were built. The stereochemical quality of the models was further analyzed using PROCHECK [22]. In the best scoring model, unfavorable protein conformations were limited to regions for which no template structures were available. In the next step, we performed MD simulations on the PLUTO model for 20-ns to measure stability of the structure. Therefore, the protein model was embedded in POPC lipid bilayer and explicit water was utilized for solvation of the membrane. During the MD simulations RMSD of TM helices C_α_ atoms from starting coordinates were monitored. Throughout the simulations RMSD of C_α_ atoms were steady and did not exceed more than 3 Å, suggesting the stability of the homology model (Figure S3).

**Figure 3:**
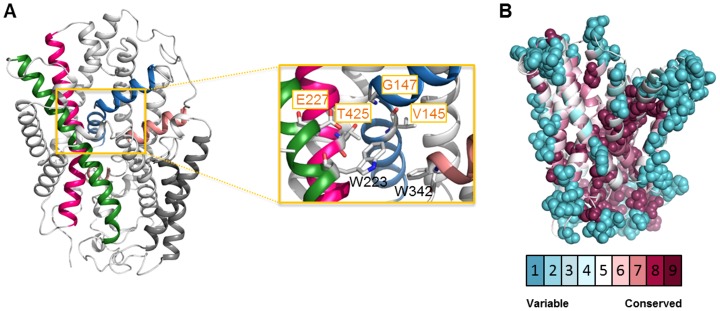
Position of functionally relevant residues in the PLUTO structural model. (A) The PLUTO homology model was built using I-TASSER server based on the open conformation of MHP1 (PDB ID: 2JLN) and its quality was assessed using the PROCHECK program. Important transmembrane segments involved in nucleobase binding are marked in blue (TMs 1a and 1b), green (TM 3), pink (TM 8) and salmon (TMs 6a and 6b). Residues directly involved in substrate binding are V145, G147, E227 and T425 (marked with yellow boxes and shown in the middle). (B) Evolutionary conservation profile of the PLUTO core protein (TM1-TM10) calculated by Consurf server [23] shows a high conservation of the substrate binding pocket. The PLUTO homology model is colored according to the conservation score (based on ClustalW matrix).

**Figure 4:**
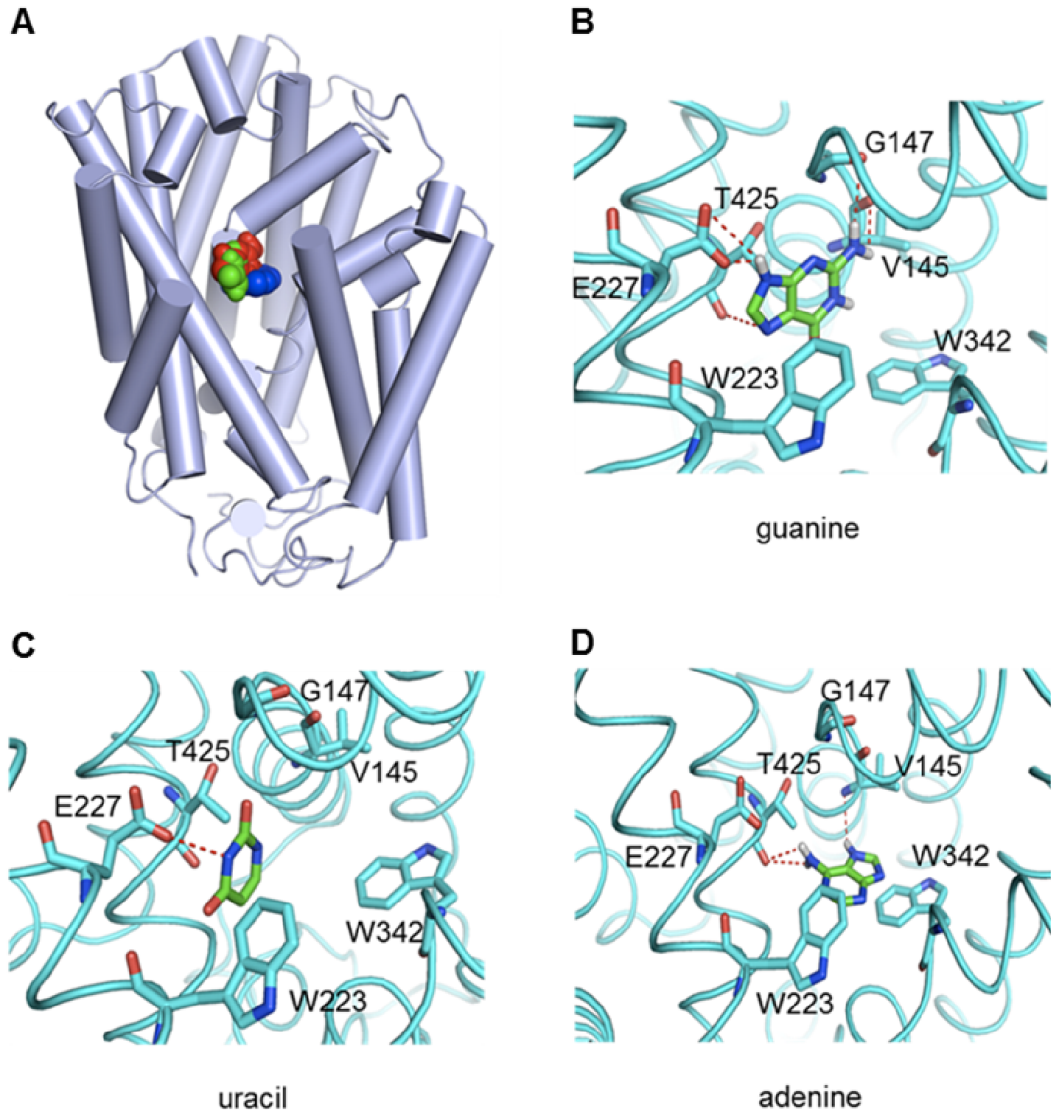
Docking poses of substrates. (A) Schematic overview of the docking poses of uracil (green), guanine (red) and adenine (blue). Uracil and guanine exhibit a similar binding mode, whereas adenine binds deep into the catalytic pocket of PLUTO. (B) Docking pose of guanine. (C) Docking pose of uracil. (D) Docking pose of adenine. Glide 5.0 (Schrodinger software) was used for docking purposes and the standard precision mode was selected to dock the substrates into the receptor site.

The structural model of PLUTO (for PDB file see File S1) is composed of 12 TM helices, in which 10 TMs form a compact core followed by two additional TMs (Fig 1B, 3A). The PLUTO structure contains the characteristic ‘5+5’ inverted structural symmetry motif defined by TM1-TM10. Within the “inverted repeat” motif, two sets of five TMs are oppositely oriented with respect to the each other around an axis in the center of the membrane and parallel to the plane. For convenience, the structure can be divided into two topologically distinct subdomains. The first is a four helix bundle comprising TMs 1 and 2 and their pseudo two-fold equivalents TMs 6 and 7 are proposed to form the substrate binding site (Figure 4A). The second is another motif of four helices formed from TMs 3 and 4 and their pseudo two-fold equivalents, TMs 8 and 9 and forms a possible ion-binding site [26]. Conserved residues are located in the core of the structure (Figure 3B). The substrate binding site is located in the center of the helical bundle (Figure 3A, 4A).

### Substrate docking

All the physiological substrates of PLUTO namely adenine, guanine and uracil were docked into the substrate binding pocket of the protein using the standard precision (SP) protocol. All of these substrates bind into a site located in the middle of the membrane-spanning regions, where they form direct contact with residues from TM1, TM3, TM6 and TM8 (Figure 3A, 4A). Residues in these regions have also been found important for ligand binding in MHP1 [26] and FCYB [8], which suggest that the fold and function of this protein region is highly conserved among the NCS1 members. This is also supported by an evolutionary conservation profile as shown (Figure 3B). Guanine interacts with PLUTO through a bidentate H bond between purine N9 and residue Glu-227 and two H bonds formed between purine C2-NH_2_ and backbone carboxyl groups of residue Val-145 and Gly-147 (Figure 4B), uracil adopts a binding mode in which pyrimidine N3 of uracil forms a H bond with residue Glu-227 (Figure 4C). While both guanine and uracil interact with Glu-227 through H bonds, in contrast adenine binds deep into the catalytic pocket of PLUTO located in a 5 Å distance from the side chain of Glu-227 (Figure 4D). Adenine interaction is stabilized by three H bonds, one between purine N7 and Val-145 and a bidentate H bond between purine C6-NH_2_ and carboxyl group of Thr-425. In addition adenine exhibited weak *pi*-stacking interaction with indole rings of Trp-342.

### Validation of docking model by further functional studies

Using docking studies as an initial guide we identified further critical residues that are located within 5 Å of the putative binding site in addition to Gly-147 and Glu-227. We substituted each position with one or more residues having different physiochemical properties of the wild-type residue. Residues in the proximity of the proposed binding pocket that were mutated in the course of this work and shown to affect transport properties are listed in Table 1 and their position is shown in Figure 1A. The transport of all the PLUTO substrates in all the single mutants, V145A, T425A, W342A, and W223A, L144A, F341A, N426A, N430A and I226A was not markedly altered (Table 1). However, interesting observations for the double mutants of critical residues were made. The single mutant T425A had no effect on the transport but when a double mutant E227Q T425A was tested, it reduced guanine and adenine transport to 25% and 32%, respectively (Figure 2B, C). In the docking pose both guanine and adenine appear to form a H bond with the backbone carboxyl group of Thr-425, however uracil does not show any interaction with Thr-425. Mutation of Thr-425 with any other amino acid residue will change the side chain only but the backbone carboxyl group will remain intact, which probably explains why the single mutant with Thr-425 had no effect on the transport. However, when the double mutant E227Q T425A was studied the effect of Thr-425 binding was more visible, especially on adenine transport, which was shown to be unaffected by the single mutants E227Q and T425A. A similar trend, although to a lesser extent was observed with double mutant V145A G147Q, which reduced guanine transport to 12% relative to controls. Interestingly, uracil transport in this mutant was even increased by 50% (Figure 2A). The mutational analysis of the putative substrate binding site revealed that uracil transport is affected by Glu-227, guanine transport by Glu-227, Thr-425, Val-145 and Gly-147 whereas adenine transport relies on Thr-425 and Gly-147.

### Residues functioning as a selectivity filter

**Figure 5:**
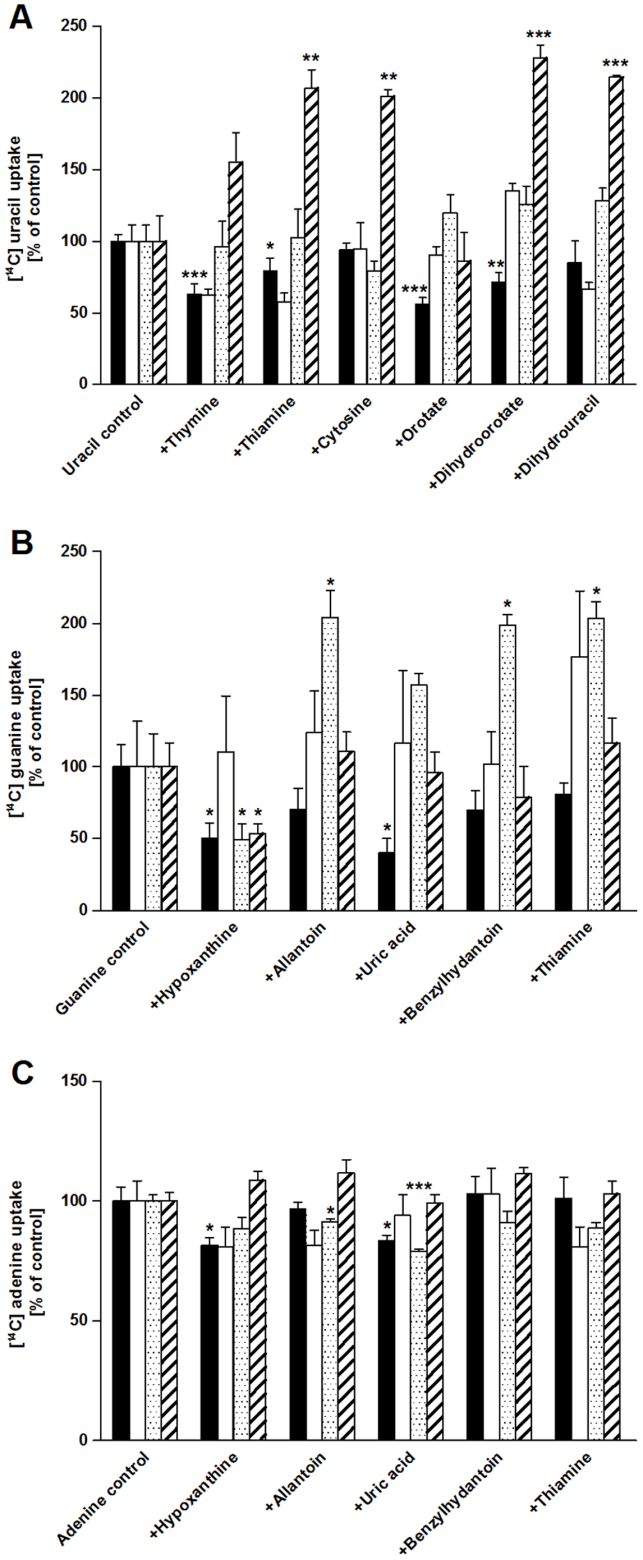
Competition studies with PLUTO mutants putatively acting as specificity filter. Direct uptake studies were performed after heterologous expression of PLUTO (black) and PLUTO mutants W223A (white), F341A (spotted) and I226A (striped) in *E. coli* cells lacking the endogenous uracil transporter uraA. The uptake was measured for uracil (A), guanine (B) and adenine (C) with concentrations of 20 μM and several competitors were added with ten-fold excess (200 μM). The data represent the mean of net uptake rates of at least three independent experiments ± SE. The asterisks indicate significant differences between PLUTO mutants and the control based on Student's t-test (*  = p<0,05; **  = p<0,01; ***  = p<0,005).

In the PLUTO model, side chains of Ile-226, Phe-341 and Trp-223 are orientated upstream of the binding site, ideally positioned for defining the entrance of substrates in a trajectory leading to the binding site. Due to the strategic location of these residues above the binding site we hypothesize that these residues might be critical for determining selectivity for the physiological substrates of PLUTO by preventing substrate analogs from entering into the binding pocket. When the residues are substituted with short side chain residues they might allow passage of substrate analogues with bulky groups. Similarly, in the uric acid-xanthine permease of *Aspergillus nidulans*, a hydrophobic residue Phe-528 located above the substrate binding site has been proven to be a molecular filter [27]. To test our hypothesis, all three residues were mutated and subsequently tested for transport function. However, no differences concerning direct uptake of radiolabelled uracil, guanine or adenine were observed (Table 1, Figure 5), suggesting they do not play a role in substrate binding.

In the second step, structurally similar substrates were tested in competition experiments. Adenine and guanine (20 μM) transport was tested in the presence of 200 μM hypoxanthine, allantoin, uric acid, benzylhydantoin, or thiamine. Uracil (20 μM) transport was tested in the presence of 200 μM thymine, thiamine, cytosine, orotate, dihydroorotate and dihydrouracil. Guanine transport in wild type PLUTO was affected by hypoxanthine and uric acid (Figure 5B) and uracil transport by thymine and orotate (Figure 5A). The latter two substances were also tested in direct uptake studies and were not identified as transport substrates. In W223A mutants, hypoxanthine and uric acid were no longer able to inhibit guanine transport (Figure 5B) while uracil transport was inhibited by thymine, thiamine and dihydrouracil (Fig 5A). In F341A and I226A mutants guanine transport was inhibited by hypoxanthine, no further effects of competing substrates were identified (Figure 5). No competitive inhibition of adenine transport was observed in any of the proteins (Figure 5C).

### Competitive binding of substrates

**Figure 6:**
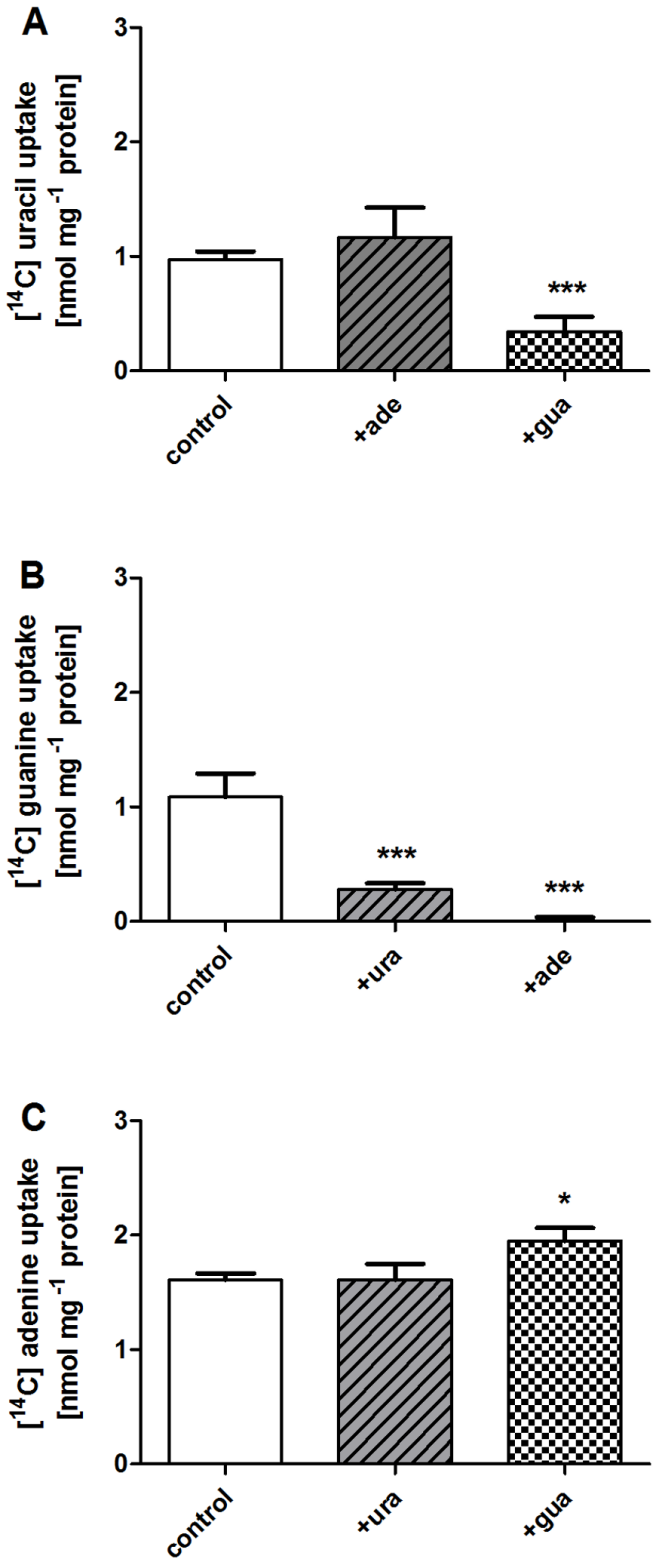
Substrate competition studies. Direct uptake studies were performed after heterologous expression of PLUTO in *E. coli* cells lacking the endogenous uracil transporter uraA. The uptake of uracil (A), guanine (B) and adenine (C) was measured after the incubation with 20 μM substrate for 2 minutes (control). To investigate the effect of the other substrates, they were added to the uptake medium with ten-fold excess (200 μM). The data represent the mean of net uptake rates of at least three independent experiments ± SE. The asterisks indicate significant differences between PLUTO mutants and the control based on Student's t-test (*  = p<0,05; ***  = p<0,005).

Our PLUTO docking and site-directed mutagenesis results suggest the binding sites for uracil and guanine overlap whereas adenine adopts a different binding mode and binds deeper into the protein cavity. Therefore, we envisioned that competition studies with the substrates would provide some additional insight into the substrate binding. To check this, competition studies with all substrates were performed. Transport of 20 μM [^14^C]-uracil was reduced by adding 200 μM guanine, but remained unaffected by the same concentration of adenine (Figure 6A). In case of guanine transport, both uracil and adenine had marked inhibitory effects (Figure 6B). Only adenine was not influenced by the presence of a ten times higher concentration of uracil or guanine (Figure 6C).

**Figure 7:**
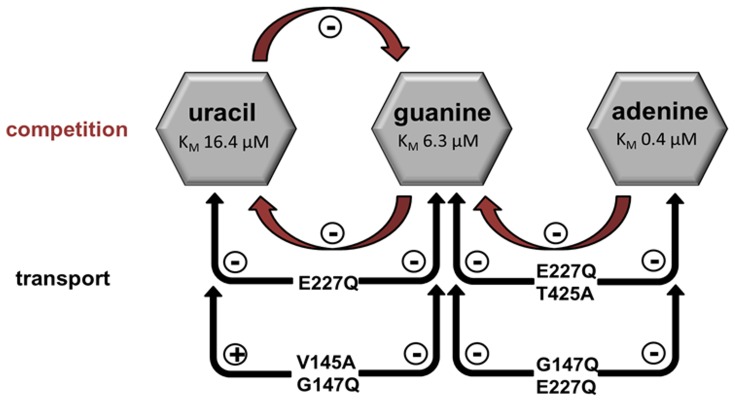
Schematic overview of competition between the different substrates of PLUTO (red) and transport activities of different PLUTO mutants (black). The presence of uracil leads to a competitive inhibition of guanine transport and vice versa. Furthermore, adenine leads to a competitive inhibition of guanine transport. The mutation E227Q leads to a loss of uracil and guanine transport actitivity (-), whereas the double mutation V145A G147Q only affects guanine transport. In both double mutants E227Q T425A and G147Q E227Q guanine and adenine transport is affected.

When combining the obvious effects of competition studies and transport experiments with mutated PLUTO protein the following conclusions can be drawn: Uracil and adenine binding pockets are independent. Apart from the drastic mutations E227A, E227K and E227Q G147Q which affect all substrates, the remaining mutants affect either uracil and guanine or adenine and guanine transport (Figure 7). The same holds true for the competition experiments. Adenine transport is only affected by the two double mutations: E227Q T425A and E227Q G147Q (Figure 7). Uracil transport is affected by the mutations E227Q, V145A G147Q and in addition by guanine as competitor (Figure 7). Guanine transport is affected by all mutations and in addition by both other substrates as competitors (Figure 7).

## Discussion

A systematic mutational analysis of residues forming the putative substrate binding pocket in PLUTO has been performed to delineate their role as structural determinants for substrate binding. In the first step, conserved amino acid residues were identified using a multiple sequence alignment of NCS1 proteins, including PLUTO and MHP1 [4]. Two out of six residues identified as putative substrate binding site in the MHP1 crystal structure differ compared to PLUTO. Besides differences in key amino acid residues, the substrates transported by PLUTO and MHP1 are different. PLUTO transports uracil, adenine and guanine, whereas MHP1 facilitates transport of 5-benzyl hydantoin and 5-indolyl-methyl hydantoin [4], [28]. When Glu-227 and Gly-147 were exchanged to those amino acids present in MHP1 in the double mutant E227Q G147Q, PLUTO substrates were no longer transported (Figure 2 A–C). Importantly, we confirmed the presence of similar amounts of protein in the double mutant and control cells by western blot on total membrane preparations from induced *E. coli* cells (Figure S1). To test whether PLUTO can transport the known substrate of MHP1 benzyl hydantoin uptake by wild-type PLUTO was studied using competitive binding analysis. However, no indication for a transport of benzyl hydantoin was obtained. These results indicate that although PLUTO and MHP1 belong to same protein family, their active sites are arranged differently, thus defining a molecular determinant for substrate selectivity.

To gain further insight into substrate binding, in a second step the homology model was used to dock all the three physiological substrates of PLUTO into the protein cavity to develop a three-dimensional model of PLUTO-substrate binding. Overall the homology model is similar to the crystal structure of the outward facing conformation of MHP1. Superimposition of all C_α_ atoms in PLUTO and MHP1 gives a RMSD of 2.7 Å. 4 TM helices including TM1, TM3, TM6 and TM8 form the ligand-binding site, in which TM1 and TM6 show unwound regions, whereas TM8 is slightly bent. A common arrangement with unwound and bent helices in the ligand binding site is observed in both, MHP1 and FCYB models. These structural conformations are important for transporter function, although one notable difference was found in the unwound region of TM6, which is more extended in PLUTO than in MHP1. We also found that some of the residues contributing to the structural organization at the ligand-binding site are not conserved between NCS1 members, such structural heterogeneity might be a prerequisite for the substrate specificity within the family members.

This information allowed us to identify key residues in ligand binding and to perform a comprehensive mutational mapping of these residues. Several important features regarding the molecular determinants of PLUTO substrate binding emerge from the study. First, the mutational analysis clearly identified Glu-227 as a critical determinant for guanine and uracil binding. Second, docking analysis identified Thr-425 in addition to Gly-147 as possible adenine interaction partner. However, although the binding of adenine is independent of Glu-227, the mutation E227Q G147Q completely abolished the transport of adenine. Third, as evidenced by the substrate competition studies and docking pose, the binding sites for uracil and guanine overlap, whereas adenine binds deeper into the cavity.

Some of the active residues showed backbone H-bonding with the substrates, however validation of such interactions is difficult by single mutations. Therefore, double mutations of these residues with E227Q were generated. E227Q was chosen because here the differences in response of uracil, guanine and adenine were most pronounced. In fact, changing Thr-425 to alanine in E227Q T425A led to highly reduced adenine and guanine transport. This fits nicely to the docking models of both purine bases, which interact with Thr-425. T425A alone showed no effect on adenine transport. This indicates that mutation of Glu-227 somehow modifies the binding pocket but the second mutation is required to restrict purine transport. V145A G147Q most markedly affects guanine transport. Both residues bind guanine according to the docking studies, whereas adenine is only interacting with Val-145 and uracil does not interact with any of the two. However, adenine transport is mainly influenced by modification of Gly-147 in close proximity to Val-145. Possibly our docking model does not allow exact prediction of H bond formation between adenine N7 and the corresponding amino acid residue.

To gain further insight into the role of residue Glu-227, the mutants E227D, E227Q, E227K and E227A were constructed and analyzed. Reduction of the acidic chain of Glu-227 by one methylene (E227D) reduced transport of uracil and guanine. At the same time the affinity towards uracil increased in this mutant (Table 2). Interestingly, a similar mutation in the uric acid/xanthine transporter UapA of *Aspergillus nidulans* E356D led to dramatically increased binding of the substrate but reduced transport [29]. Glu-227 is located in the middle of TM3, thereby residing between substrate binding-site and co-ion binding-site and possibly connecting these two sites. Strategic location of Glu-227 might allow the residue to play a dual role in both substrate and ion binding, thus becoming a key element in coupling. A dual role of Glu-269 in both substrate and ion binding has already been observed in LacY permease [30], [31]. Although LacY is only distantly related to the bacterial nucleoside transporter NupG, both share common mechanistic features, as recently shown [32]. Moreover, Glu-264 aligning close to Glu-269 in LacY was shown to be important for uridine transport in NupG [32]. Probably due to its dual role in PLUTO, Glu-227 coordinates between ion and substrate binding. Once the residue is substituted with aspartate such link is broken, which might explain why E227D in spite of having higher substrate affinity showed reduced transport. Adenine transport is different, as the affinity of PLUTO is much higher with a K_M_ value of 0.38 μM. Although the affinity drops in E227D to 0.97 μM this is still 7–10 times higher compared to uracil or guanine. The high affinity of PLUTO for adenine is probably one reason for the low effect of mutations and competitors on adenine transport (Figure 5C, 6). In general, Glu-227 and Gly-147 mutants showed no obvious alterations in their substrate affinities.

Recently, the purine/cytosine transporter FCYB from *A. nidulans* was analyzed by modeling, docking and mutational studies [8]. Like PLUTO and MHP1, FCYB is a member of the NCS1 family. In FCYB five amino acid residues were identified as critical for substrate binding, these are Ser-85, Trp-159, Asn-163, Trp-259 and Asn-354. These align to Gly-147, Trp-223, Glu-227, Trp-342 and Asn-430 in the PLUTO sequence, respectively (Figure 1A). All of these PLUTO residues were mutated in the course of this work. One can differentiate between mutants directly involved in substrate binding (Ser-85, Asn-163, and Asn-354) and residues with a function in stabilizing substrate binding by pi-stacking interactions (Trp-159 and Trp-259) [8]. With respect to direct binding, only Glu-227 single mutants markedly affected the PLUTO transport capacity, equivalent to Asn-163 in FCYB. Single mutants of the other residues Gly-147 and Asn-430 showed only weak or no effects (Table 1). The tryptophan residues involved in *pi*-stacking interaction in FCYB are also conserved in PLUTO. However, due to an antiparallel orientation in PLUTO, different to the situation in FCYB, the *pi*-stacking interactions are weak and no effect on PLUTO facilitated transport of any substrate was identified in corresponding mutants. Thus, these interactions seem to be of minor importance for PLUTO function.

It was concluded that residues corresponding to Trp-159, Trp-259 Asn-350, Pro-353 and Asn-354 in FCYB are major elements of the substrate binding site in all NCS1 members [8]. As discussed above this does not hold true for both tryptophan residues in PLUTO. Asn-350 equivalent to Asn-426 in PLUTO was also mutated in the course of this work and shown to have no effect on transport. Furthermore, two additional residues Val-145 and Thr-425 were identified as important components of the substrate binding site. These results highlight the difference between the substrate binding properties of PLUTO and FCYB. Moreover, Asn-430 in PLUTO is highly conserved in NCS1 members and mutations extinguish the transport capacity of FCYB, but not of PLUTO [4], [8].

Asn-163 is conserved in nearly all purine specific FCY-like NCS1 proteins and is replaced by Glu-227 in PLUTO [4], [8]. When this residue was mutated to Leu and Gln the transport capacity of FCYB was highly reduced or lost. Similarly, replacing Glu-227 by Gln exerted the strongest observed reduction in PLUTO mediated transport. Only Glu-227 seems to allow for a high capacity of purine and pyrimidine nucleobase transport of PLUTO. Interestingly, the yeast uracil transporter FUR4 carries a Gln residue at this position. Obviously, the important function of residue 227 awaits further clarification.

All FCYB substrates, adenine, hypoxanthine, guanine and cytosine interact with the same residues and a simple model of competition between substrates and the binding site is assumed [33]. This situation is obviously different in PLUTO where the substrates interact with different residues and thus show different responses in competition experiments.

A further aim was to identify the molecular basis of substrate selectivity using competition studies of functional mutants towards a plethora of substrates. In the PLUTO model Ile226, Phe341 and Trp223 are located above the binding site, which led to speculations about further roles of these residues in substrate selection. Substitution of the above mentioned residues with alanine does not affect transport and the specificity profile of PLUTO for its physiological substrates, suggesting that these residues are not an essential part of the substrate binding site. Among these mutants, W223A showed most dramatic results, as competition inhibition profiles of both uracil and guanine for non-physiological substrates were altered in the W223A mutant. Thus, Trp223 might be involved in the ‘selection’ of physiological substrates by performing *pi-pi* interactions with particular substrates and not with others, depending on how substrates are orientated in the nearby binding site. Furthermore, Trp117 in MHP1, equivalent to Trp223 in PLUTO, showed significant side chain movement during transition between outward to substrate-bound occluded conformation, indicating its role in either substrate binding or selection [18]. However the competition studies with mutant W223A do not provide strong evidence for a role in substrate-selectivity but might be useful for further investigations about the molecular basis of substrate selection in PLUTO and NCS1 members in general.

From the results obtained in uptake and competition studies as well as docking analysis, a model was generated (Figure 7). From this model, it can be concluded that guanine transport is most strongly affected by mutations and competes with uracil and adenine. This reflects the fact that guanine shares common amino acid residues with uracil (E227) and with adenine (T425 and G147) binding pockets. Furthermore, inhibition of guanine binding by excess of adenine can be due to the relatively large size of both substrates which could lead to steric clashes during the binding and eventually reduce the transport. Uracil only interacts with guanine as it shares no interacting amino acid residues with adenine. Finally, adenine is unaffected by competing substrates as it is located at a different position within the substrate binding pocket and exhibits the highest substrate affinity.

From the competition studies with purine related compounds, hypoxanthine was identified as a putative further substrate as it shows marked inhibition of guanine transport. Whereas the physiological function of uracil import into plastids is quite clear, it is unknown whether adenine, guanine or hypoxanthine might be further processed in this compartment. As discussed elsewhere, it is unlikely that adenine recycling to AMP by adenine phosphoribosyl-transferase (APRT) takes place in plastids [6]. Remarkably, mutants impaired in adenine recycling due to a mutation in the main APRT isoform showed a 50% increase in intracellular adenine levels accompanied by improved growth and stress tolerance [34]. The observed stress tolerance was attributed to a signaling function of adenine. However, it was not clarified in which intracellular compartment such signaling was exerted. It was shown that guanine is recycled to GMP by hypoxanthine/guanine phosphoribosyltransferase which represents a single copy gene in Arabidopsis. Corresponding knockout mutants were almost completely resistant to toxic 8-azaguanine during germination which is in line with the inability of such mutants to incorporate 8-azaguanine into RNA and DNA, leading to its toxicity [35]. There is no indication that this enzyme or Arabidopsis APRTs are located to plastids by bioinformatic analysis [6] or analysis of corresponding mutants [35] nor is the catabolism of purine nucleobases located in plastids [36].

## Conclusion

In conclusion, it was shown that by combining structural and functional studies including mutants, critical amino acid residues involved in interaction with the different substrates of PLUTO could be identified. By this, a homology model constructed on the basis of the structural information from MHP1 was validated. Significant differences in structure function relations between PLUTO and the only other characterized eukaryotic member of the NCS1 family, FCYB were identified. Although the PLUTO structural model predicts a single binding site, not all substrates compete with each other due to minor differences in the interaction with specific amino acid residues. This illustrates that competition studies often cannot replace direct transport studies. Another prominent example for this is the sucrose transporter SUC5 facilitating sucrose and biotin transport without reciprocal inhibition [37]. These results highlight key differences between active site architecture of PLUTO and NCS1 family members, which will be important for the prediction of the substrate specificity in other NCS1 proteins.

## Supporting Information

Figure S1 **Confirmation of protein content in** ***E. coli*** **cells expressing PLUTO and PLUTO mutants.** To check for the presence of PLUTO in control cells and in the mutants E227K, E227Q G147Q, E227A, E227Q, E227D, G147Q, V145A G147Q and E227Q T425A, membranes were isolated from wildtype and mutated *E. coli* cells with (+) or without induction (−). 5 μg (A) and 10 μg (B) of protein were spotted on a membrane and developed with anti MAT-Tag antibody.
(TIFF)

Figure S2 **Nucleobase uptake with and without MAT-Tag.** The influence of a C-terminal MAT-Tag was tested with direct uptake studies of uracil, adenine and guanine after PLUTO expression with and without MAT-Tag in *E. coli* cells lacking the endogenous uracil transporter uraA. The cells were incubated with radiolabeled nucleobases (20 μM) for 2 minutes. The data represent the mean of net uptake rates of 3 independent experiments ± SE and uninduced cells were used as a control.
(TIFF)

Figure S3 **RMSF and RMSD values for PLUTO homology model.** (A) The RMSF curve for PLUTO Homology model at 20ns MD simulation. (B) The RMSD curve for PLUTO Homology model along 20ns MD simulation. The initial equilibration steps were skipped.
(TIFF)

File S1 **PLUTO Homology model.**
(PDB)

Table S1 **Primers used in this study.**
(PDF)
