# Supplementary figures and images for: Structure-Function Relationship of a Plant NCS1 Member – Homology Modeling and Mutagenesis Identified Residues Critical for Substrate Specificity of PLUTO, a Nucleobase Transporter from Arabidopsis
Source: PLoS One. 2014 Mar 12;9(3):e91343. doi: 10.1371/journal.pone.0091343 (PMC3951388; doi:10.1371/journal.pone.0091343)

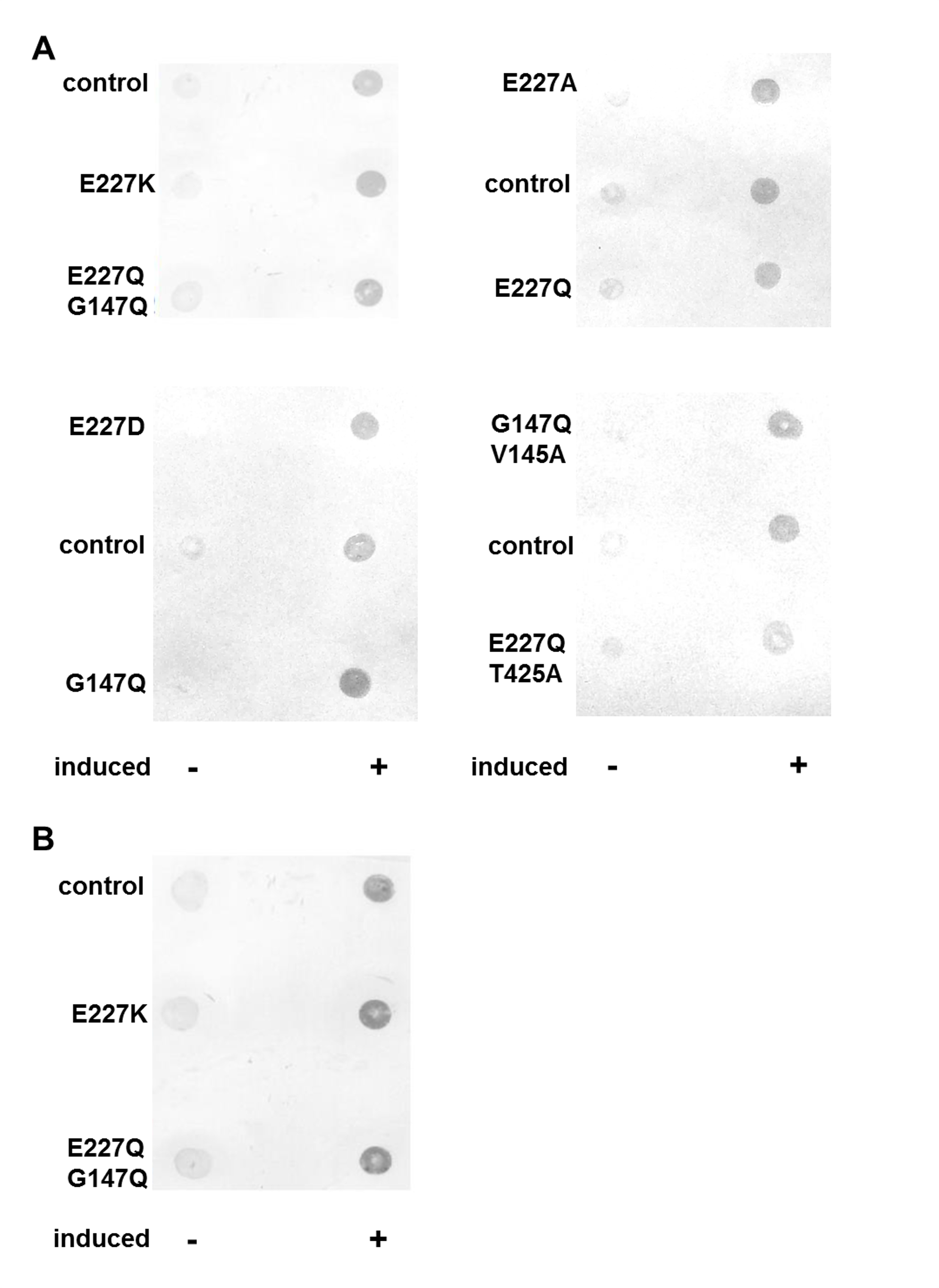

Supplement: Figure S1 — Confirmation of protein content in E. coli cells expressing PLUTO and PLUTO mutants. To check for the presence of PLUTO in control cells and in the mutants E227K, E227Q G147Q, E227A, E227Q, E227D, G147Q, V145A G147Q and E227Q T425A, membranes were isolated from wildtype and mutated E. coli cells with (+) or without induction (−). 5 μg (A) and 10 μg (B) of protein were spotted on a membrane and developed with anti MAT-Tag antibody. (TIFF) [file pone.0091343.s001.tif]

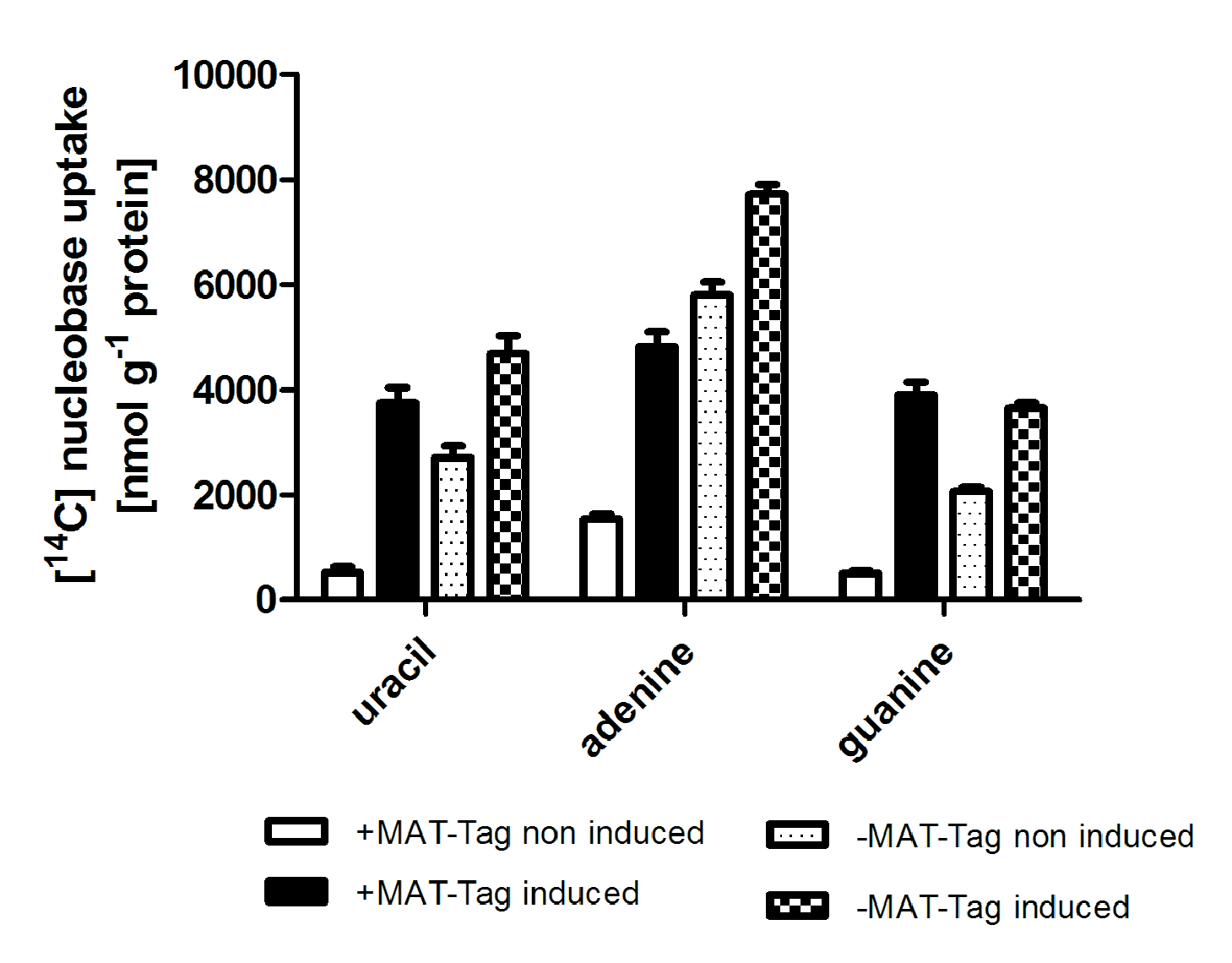

Supplement: Figure S2 — Nucleobase uptake with and without MAT-Tag. The influence of a C-terminal MAT-Tag was tested with direct uptake studies of uracil, adenine and guanine after PLUTO expression with and without MAT-Tag in E. coli cells lacking the endogenous uracil transporter uraA. The cells were incubated with radiolabeled nucleobases (20 μM) for 2 minutes. The data represent the mean of net uptake rates of 3 independent experiments ± SE and uninduced cells were used as a control. (TIFF) [file pone.0091343.s002.tif]

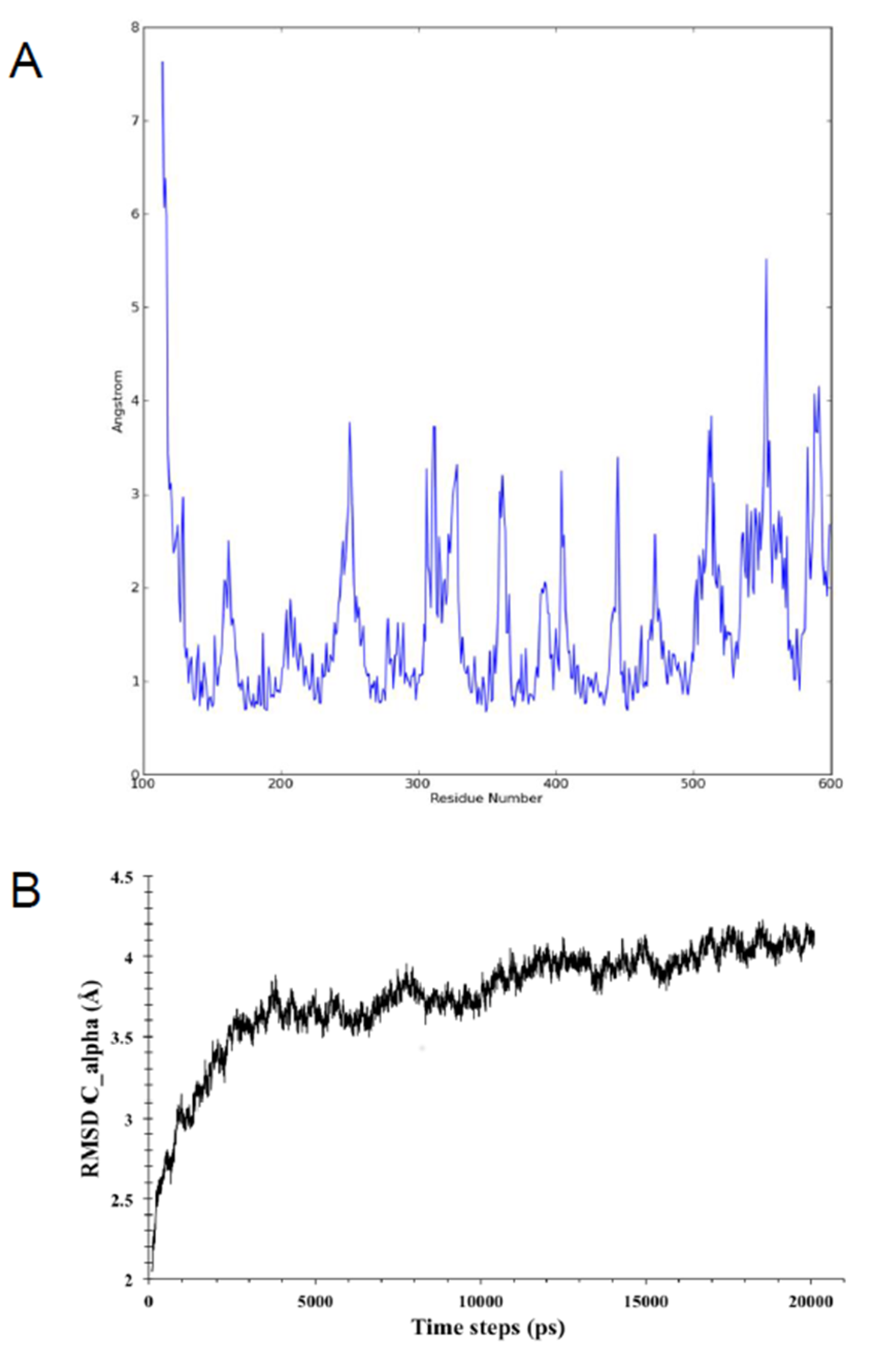

Supplement: Figure S3 — RMSF and RMSD values for PLUTO homology model. (A) The RMSF curve for PLUTO Homology model at 20ns MD simulation. (B) The RMSD curve for PLUTO Homology model along 20ns MD simulation. The initial equilibration steps were skipped. (TIFF) [file pone.0091343.s003.tif]
