# Supplementary material for: Structure-Function Relationship of a Plant NCS1 Member – Homology Modeling and Mutagenesis Identified Residues Critical for Substrate Specificity of PLUTO, a Nucleobase Transporter from Arabidopsis
Source: PLoS One. 2014 Mar 12;9(3):e91343. doi: 10.1371/journal.pone.0091343 (PMC3951388; doi:10.1371/journal.pone.0091343)
Supplement: Table S1 — Primers used in this study. (PDF) [file pone.0091343.s005.pdf]

Table S1. Primers used in this study.

**<u>Primers for heterologous PLUTO expression in *Escherichia coli*</u>**	
PLUTO_pTACMAT2_fwd	5‘-TTTCTCGAGTGGTCTCCAATTGCTTAAGC-3‘
PLUTO_pTACMAT2_rev	5‘-TTTGGTACCAAAGCGGATGTGAAGAAG-3‘
**<u>Primers for PLUTO Mutagenesis</u>**	
PLUTO_E227Q_sense	5‘-CGGATGGTATGGTATCCAGACATGGATCGGAGG-3‘
PLUTO_E227Q_antisense	5‘-CCTCCGATCCATGTCTGGATACCATACCATCCG-3‘
PLUTO_E227A_sense	5‘-GGATGGTATGGTATCGCGACATGGATCGGAGGT-3‘
PLUTO_E227A_antisense	5‘-ACCTCCGATCCATGTCGCGATACCATACCATCC-3‘
PLUTO_E227D_sense	5‘-CGGATGGTATGGTATCGATACATGGATCGGAGGT-3‘
PLUTO_E227D_antisense	5‘-ACCTCCGATCCATGTATCGATACCATACCATCCG-3‘
PLUTO_E227K_sense	5‘-CGGATGGTATGGTATCAAGACATGGATCGGAGG-3‘
PLUTO_E227K_antisense	5‘-CCTCCGATCCATGTCTTGATACCATACCATCCG-3‘
PLUTO_G147Q_sense	5‘-GTCAAGTTTATGGATCGGTCTCGTAGTTCAGGTGCCTACTTACTAC-3‘
PLUTO_G147Q_antisense	5‘-GTAGTAAGTAGGCACCTGAACTACGAGACCGATCCATAAACTTGAC-3‘
PLUTO_G147K_sense	5‘-ATGTCAAGTTTATGGATCGGTCTCGTAGTTAAGGTGCCTACTTACTACC-3‘
PLUTO_G147K_antisense	5‘-GGTAGTAAGTAGGCACCTTAACTACGAGACCGATCCATAAACTTGACAT-3‘
PLUTO_W342A_sense	5‘-CTAACCGCAAACATAAGCTTCGCGGCAACTCTAGCTTTAAACAT-3‘
PLUTO_W342A_antisense	5‘-ATGTTTAAAGCTAGAGTTGCCGCGAAGCTTATGTTTGCGGTTAG-3‘
PLUTO_V145A_sense	5‘-TTTATGGATCGGTCTCGCAGTTGGTGTGCCTACTT-3‘
PLUTO_V145A_antisense	5‘-AAGTAGGCACACCAACTGCGAGACCGATCCATAAA-3‘
PLUTO_T425A_sense	5‘-CCCTAGCCACACTCACTGCAAACATAGCTGCAAAC-3‘
PLUTO_T425A_antisense	5‘-GTTTGCAGCTATGTTTGCAGTGAGTGTGACTAGGG-3‘
PLUTO_W223A_sense	5‘-GCATTAGTCGGTTGCGGAGCGTATGGTATCGAGACATG-3‘
PLUTO_W223A_antisense	5‘-CATGTCTCGATACCATACGCTCCGCAACCGACTAATGC-3‘
PLUTO_L144A_sense	5‘-ATGTCAAGTTTATGGATCGGTGCCGTAGTTGGTGTGCCTAC-3‘
PLUTO_L144A_antisense	5‘-GTAGGCACACCAACTACGGCACCGATCCATAAACTTGACAT-3‘
PLUTO_F341A_sense	5‘-CCTAACCGCAAACATAAGCGCCTGGGCAACTCTAGCTTTA-3‘
PLUTO_F341A_antisense	5‘-TAAAGCTAGAGTTGCCCAGGCGCTTATGTTTGCGGTTAGG-3‘

PLUTO_N426A_sense	5‘-CCTAGCCACACTCACTACAGCCATAGCTGCAAACGTGGTC-3‘
PLUTO_N426A_antisense	5‘-GACCACGTTTGCAGCTATGGCTGTAGTGAGTGTGGCTAGG-3‘
PLUTO_N430A_sense	5‘-CACTACAAACATAGCTGCAGCCGTGGTCGCCCCAG-3‘
PLUTO_N430A_antisense	5‘-CTGGGGCGACCACGGCTGCAGCTATGTTTGTAGTG-3‘
PLUTO_V145A_G147Q_sense	5‘-CAAGTTTATGGATCGGTCTCGCAGTTCAGGTGCCTACTTACTACCTCGCC-3‘
PLUTO_V145A_G147Q_antisense	5‘-GGCGAGGTAGTAAGTAGGCACCTGAACTGCGAGACCGATCCATAAACTTG-3‘
PLUTO_I226A_sense	5’-CGGTTGCGGATGGTATGGTGCCGAGACATGGATCG-3‘
PLUTO_I226A_antisense	5‘-CGATCCATGTCTCGGCACCATACCATCCGCAACCG-3‘
